# Further Development and Validation of a Measure of Compassionate Healthcare in Action

**DOI:** 10.1111/hex.70796

**Published:** 2026-08-03

**Authors:** Samantha R. Allen, Ellen Huish, Roxanne M. Parslow, Elizabeth Marks, Lucy A. Clarkson, Sarah Howard, Lucy Maddox

**Affiliations:** ^1^ University of Bath Bath Somerset UK; ^2^ Centre for Academic Primary Care University of Bristol Bristol South West England UK

**Keywords:** care, compassion, measure, metric, questionnaire, reliability, validity

## Abstract

**Background:**

Compassionate Healthcare In Action (CHIA) is a new questionnaire to measure patient experiences of compassionate care, with a particular focus on observable behaviours. It has been designed to address gaps in currently available metrics. It is generalisable across healthcare contexts and has been developed with patient and public involvement throughout. The present study sought to build on earlier development to evaluate the CHIA's face validity, perform item reduction, establish dimensionality, validity and reliability.

**Methods:**

Face validity was assessed via cognitive interviews with UK healthcare service users. Interviews used a Three‐Step Test Method to understand how CHIA items were used, and which items were considered most/least important in measuring patient experiences of compassionate care (Study 1). Cross‐sectional survey data were analysed using inter‐item correlations, item discrimination indexes, item and local dependency analyses and exploratory factor analysis. Statistical analyses were combined with cognitive interview data and panel reviewed to inform item reduction (Study 2). A final cross‐sectional survey corroborated single‐factor dimensionality through confirmatory factor analysis alongside establishing reliability and validity (Study 3).

**Results:**

21 cognitive interviews established face validity and identified items with stronger face validity (Study 1). These results were combined with survey analyses (*n* = 311). Seven items were removed resulting in a 14‐item CHIA demonstrating a single factor structure (Study 2). In Study 3, further survey analysis (*n* = 301) reinforced results showing a strong unidimensional structure, internal consistency, test‐retest reliability and validity: content, face, convergent and discriminant.

**Conclusions:**

The CHIA is a brief and generalisable metric that evaluates patient perceptions of compassionate care they receive. It has been robustly developed and has strong psychometric properties. The CHIA shows promise for supporting healthcare services to evaluate and improve their care. Further research could examine the CHIA in different contexts and populations.

**Patient or Public Contribution:**

Lived experience consultants supported prior CHIA development papers. In this paper a lived experience consultant (experience of physical and mental healthcare services) was involved in reviewing methodology and materials, advising on and helping with recruitment, contributing to review panel discussions, reviewing and authoring this manuscript. Additionally, methods throughout sought to amplify the voices of diverse populations who could complete the CHIA.

## 1 Introduction

### 1.1 Compassion in Healthcare

Compassion is a core tenet of healthcare values [1] and a fundamental part of patient‐centred care [2]. Compassion involves empathy and action: (1) emotionally understanding/connecting with patients' distress and (2) taking meaningful action to try and ease that distress [3, 4]. Models of compassion include Gilbert's model of Compassion Focussed Therapy (CFT) [5], which reaffirms compassion as involving: (1) engagement and (2) compassionate action, and Dewar and Nolan's [6] model of compassionate care which describes 'caring conversations' comprising knowing what matters to patients, understanding patients feelings and collaborating to shape outcomes.

Compassionate care is linked to multiple positive patient and staff outcomes. Specifically, patients report increased hope, wellbeing, responsibility for their health, trust and recovery alongside reduced suffering, anxiety, distress, symptoms and readmission [7, 8, 9, 10]. It is also linked to increased job satisfaction and engagement in clinicians alongside increased ability to encourage patient disclosure and treatment adherence [10]. Conversely, a lack of compassionate care is linked to increased patient frustration, overwhelm and dehumanisation, leading to a lack of dignity and hope [8, 11].

Interventions to build compassionate care in healthcare staff are mixed, some showing promising results and others no change (linked to systemic barriers/cultures) [3]. A key critique of interventions is the lack of valid and reliable ways to measure compassionate care [3]. Interventions are crucial, but difficult to deliver and assess without clear appraisal of what compassionate care looks like. Thus, metrics are essential to interventions and service improvement [3]. It is important that compassionate care metrics are robustly validated to ensure they are useful and do not inadvertently become administrative barriers to compassionate care.

Part of high‐quality validation involves prioritising the patient perspective. The purpose of Patient‐Reported Outcome Measures (PROMs) and Patient‐Reported Experience Measures (PREMs) are to capture the patient perspective. Including patient perspectives in development ensures metrics are patient‐centred and relevant; identifying symptoms, experiences and impacts that matter to patients rather than clinicians' or researchers' assumptions [12, 13]. Including the patient voice is linked to stronger content and face validity [13]. Involvement in item generation and refinement leads to clearer, more accurate and acceptable questionnaires, more likely to be completed [14]. Measures developed this way capture outcomes and experiences patients value and aid service improvement and decision‐making [12]. Guidelines for PROM development encourage patient inclusion in all stages [15], yet reviews highlight less than 7% of PROMs include patients in all development stages and 26% have no patient involvement [13]. Within compassion literature one review found compassionate care studies 'fail to adequately incorporate the understanding and experiences of patients' [10]. Recent updates showed improvement, but research still didn't balance patient and clinician samples [8].

### 1.2 Measuring Patient Experiences of Compassionate Care

Whilst multiple metrics attempt to address the measurement gap, existing measures have limitations in conceptualisation, application, validation and co‐creation. Not all development stages have been studied/reported for existing metrics (review by Sinclair et al. [4]). Limitations were reviewed in an earlier paper [16] but a summary is provided below and in Table 1.

**Table 1:** Existing patient‐reported experience measures of compassionate care.

Measure	Conceptual clarity	Subjective vs. behavioural items	Healthcare contexts	Patient involvement during development
Compassionate Care Assessment Tool (CCAT) [17]	Definition includes compassion & spiritual needs ‐ “A sympathetic consciousness of others' distress with a desire to alleviate it” and considers “compassion in the spiritual context”	Majority subjective	Acute hospitals, measure refers to “nurse”	Item generation: only used previous measures Item refinement: pilot study (*n* = 110) in one faith‐based acute hospital ‐ participants rated items
[Unnamed physician compassion scale] [7]	Definition uses only compassion ‐ “A sympathetic concern for the suffering of another, together with the inclination to give aid or support or to show mercy”	Mix of subjective & behavioural	Female breast cancer patients, measure refers to “physician”	Item generation & refinement: supported by previous qualitative research but details not provided
Schwartz Center Compassionate Care Scale (SCCCS) [18]	Definition uses only compassion ‐ “Compassionate care is when physicians, nurses and other caregivers recognise and validate the concerns, pain, distress or suffering of patients and their families and take action to address them”	Majority behavioural	Acute hospitals, measure refers to “doctor"	Item generation: committee of 20 patients & carers developed criteria Item refinement: 5 focus groups (patients, nurses, physicians)
Consultation And Relational Empathy (CARE) [19]	Definition focusses on empathy & compassion ‐ “Relational empathy is: the ability to (i) understand the patient's situation, perspective and feelings (and their attached meanings); (ii) to communicate that understanding and check its accuracy; and (iii) to act on that understanding with the patient in a helpful (therapeutic) way” Scale also uses circular items ‐ e.g. “Showing care and compassion”	Majority behavioural	General medicine/primary care, measure refers to “Doctor“ and “consultation“	Item generation: “supported by our previous qualitative work on patient views“ but details not provided Item refinement: 3 pilot studies with GP patients. Also pilots with 20 clinicians and 20 researchers
Five‐item tool to measure patient assessment of clinician compassion (TMPACC) [20]	Definition uses only compassion ‐ “An emotional response to another's pain and suffering involving an authentic desire to help”	All subjective ‐ no action orientated items	Hospital outpatients, measure refers to “your provider” (“physicians, advanced nurse practitioners, physician assistants”).	Item generation: literature review, then items rated by 4 professionals Item refinement: pilot study in an outpatient clinic (*n* = 3325)
Sinclair Compassion Questionnaire (SCQ) [21]	Definition uses only compassion ‐ “A virtuous response that seeks to address the suffering and needs of a person through relational understanding and action”	Majority subjective	Individuals with life‐limiting illnesses in either: acute care, hospice, long‐term care or homecare	Item generation: drew on previous studies such as a patient model of compassion & qualitative studies as well as a literature review Item refinement: Delphi & cognitive interviews

First, only two of the six available metrics use mostly behavioural items. The SCCCS [18] and CARE [19] both use action‐orientated items alongside more subjective items. The other metrics contain majority subjective or ambiguous questions/items. The TMPACC [20] has no action items, only using subjective ratings of experiences. Subjective items by nature are more vulnerable to variation in interpretation which could lead to less consistency in patient scores. This can make them a less practical gauge of patient perceptions. Recent research has also highlighted the importance of identifying the actions practitioners do which patients experience as compassionate [22]. The Compassionate Healthcare In Action (CHIA) metric presented in this paper addresses this gap by focusing on behaviours healthcare professionals do which impact patient experiences of compassionate care.

Second, the measures display limitations in their application across healthcare contexts. None are validated in mental healthcare and nearly all focussed on a narrow group of professionals (nurses, doctors). The physical healthcare contexts validated for are often highly specific. For example, the SCQ [21] is validated for life limiting illnesses, the SCCCS [18] and CCAT [17] for acute hospitals, and the unnamed physician compassion scale [7] for female breast cancer patients. As difficulties with compassionate care have been well documented in mental health services [23], it is important that a generalisable measure of the patient experience of compassionate care has been validated with a broader sample of users of healthcare services including mental health services as well. The current study aimed to address this limitation by recruiting people across multiple healthcare contexts.

Thirdly, there are concerns around conceptual clarity. For example, some metrics include multiple constructs (CCAT [17] ‐ compassion and spiritual needs; CARE [19] ‐ compassion and empathy), others do not use action‐orientated elements of compassion (TMPACC [20]) or use circular items (CARE [19]). Concerns with conceptual clarity can make it unclear what is measured (making score interpretation hard) or can mean measuring the wrong thing. The CHIA addresses this by focussing on one concept (patient experience of compassionate care) defined carefully and inductively in a clear development process.

Finally, only two metrics explicitly report qualitatively involving patients in item generation and refinement (SCCCS [18], SCQ [21]). Two refer to previous qualitative work but provide no detail (Unnamed physician compassion scale [7], CARE [19]). Two do not detail any qualitative patient involvement (CCAT [17], TMPACC [20]). No metrics report including patients with experience of mental health care in design or validation. Limited involvement of patient perspectives undermines the patient‐centredness of metrics, their content and face validity which can adversely impact acceptance and usability. The CHIA addresses this by demonstrating patient involvement throughout design and development to ensure it is a patient‐centred experience questionnaire.

Overall, although many compassionate care measures exist, they have limitations to their conceptual clarity, use of behavioural components, application across healthcare contexts, or lack patient involvement during development [4, 16, 24] (Table 1). Consequently, this paper describes the final development of the CHIA measure which seeks to address these gaps. The CHIA is a patient experience measure and has actively sought to include the patient perspective throughout development (item generation, refinement and validation). It seeks to provide a conceptually clear metric focusing on action‐orientated items that have been developed and validated across a broad array of healthcare contexts and personnel.

### 1.3 CHIA Questionnaire Development

**Figure 1:**
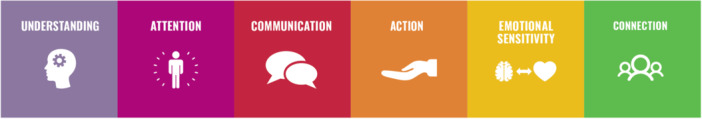
Six themes from prior item development work.

Following best practice guidelines by Boateng et al. [24] in developing and validating questionnaires, the CHIA's development involved multiple stages grouped into three phases: (1) Item Development, (2) Scale Development, (3) Scale Evaluation. Item development is reported in a previously published paper by Chatburn et al. [16], which inductively conducted key informant interviews, a literature review and Delphi. Chatburn et al.'s [16] paper resulted in 21 candidate items across six domains: Understanding, Attention, Communication, Action, Emotional Sensitivity and Connection (Figure 1). Subsequent work following Chatburn et al. [16] but prior to the current paper focussed on item reduction [25] and reduced items from 21 to a draft questionnaire comprising six items. However, feedback from multiple healthcare professionals and academic experts questioned the face validity and recommended further patient review.

### 1.4 Aims

**Figure 2:**
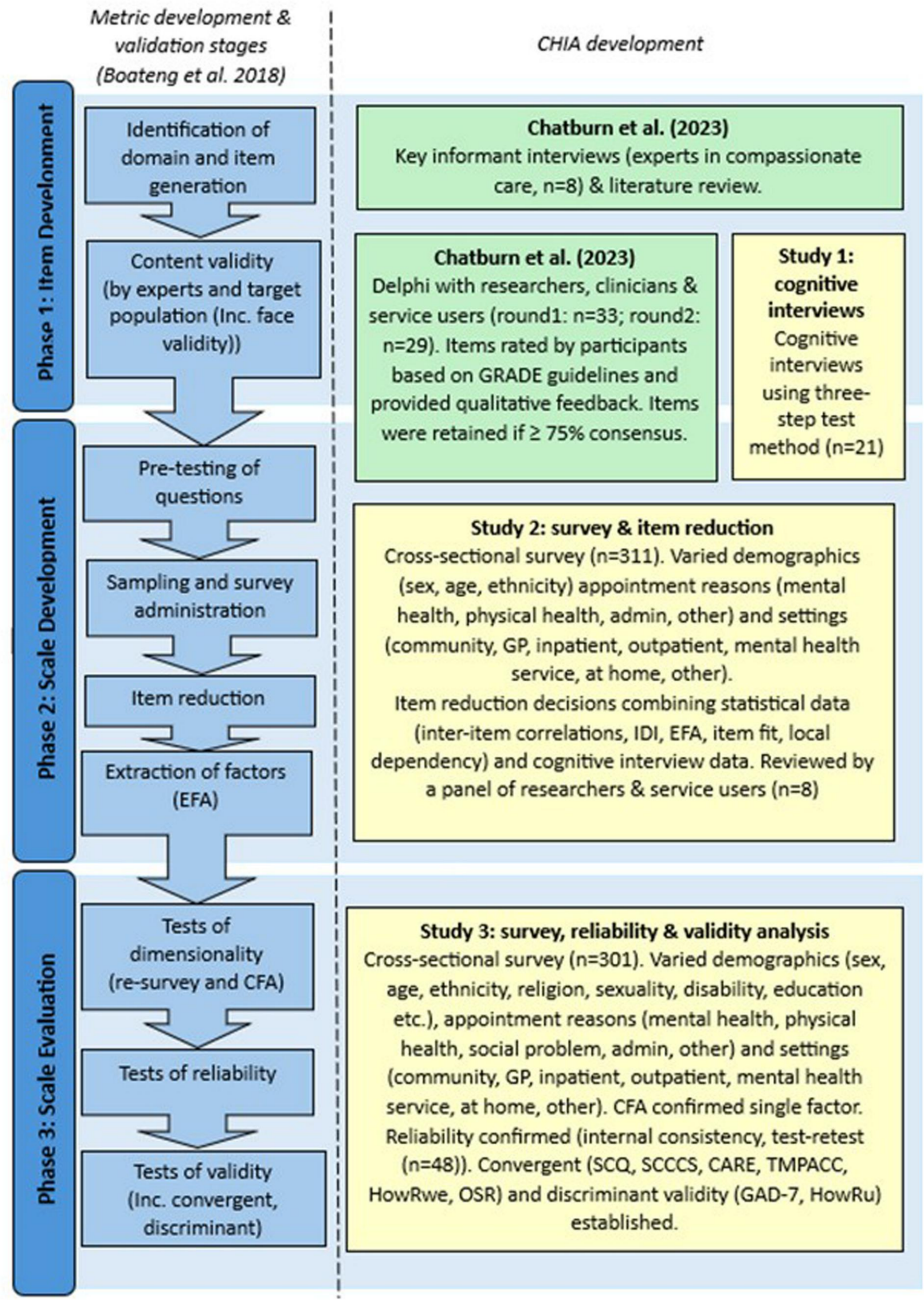
Compassionate Healthcare In Action (CHIA) development and validation across studies. *Left section of the diagram is copyright © 2018 Boateng, Neilands, Frongillo, Melgar‐Quiñonez and Young. This is reproduced with permission under the terms of the Creative Commons Attribution License (CC BY).

This multi‐study paper follows best practice for item reduction decisions and aimed to combine face validity analysis (qualitative interviews, Study 1) [24, 26] with quantitative item reduction analysis (Study 2) [24] to refine the questionnaire into its final length and item combination. The resulting final version of the CHIA was then evaluated to assess its psychometric properties (Study 3) including dimensionality, reliability and validity. Huish et al.'s [25] original dataset was re‐analysed for Study 2 and a new dataset gathered for Study 3. This multi‐study paper presents part of phase one, phases two and three of the measure development process (Figure 2). Patient involvement was key to the measures development so far and this paper sought to continue this to ensure the CHIA reflects patient experience.

This study was ethically approved by The University of Bath Social Sciences Research Ethics Committee.

### 1.5 Patient or Public Contribution

Patient involvement was integral to the CHIA's design. The previously published paper included lived‐experience consultants in CHIA development and ensured people with lived experience (alongside clinical experts) were well represented in key informant interviews and the Delphi [16], comprising 41% of the sample.

The present paper involved an individual with lived experience (involved in the previous study) as a consultant and co‐author. The consultant has experience of mental health difficulties and accessing mental and physical health services alongside a strong record of research co‐creation and contribution. Her role involved reviewing materials, interviews and surveys for accessibility, advice and help with recruitment, contribution to review panel discussions during item reduction, and review and contribution to this manuscript (summary in GRIPP2 table, Supporting Information: Appendix 1). Additionally, the cognitive interview phase (Study 1) actively sampled on the upper end of recommended ranges to encourage a wide array of patient contributions for consideration alongside statistical analyses.

## 2 Study 1: Establishing Face Validity

### 2.1 Research Questions

a. Do individual items have face validity?
b. Does the scale have face validity?

### 2.2 Materials and Methods

#### 2.2.1 Design

Study 1 followed the commonly used qualitative Three‐Step Test Interview design [27, 28, 29]. It involves three phases during cognitive interview; (1) think‐aloud (participants verbalising thought process during metric completion), (2) researcher follow‐up probing for clarification, (3) debrief to gather participants' overall experiences of the metric [27].

#### 2.2.2 Recruitment

Inclusion criteria were UK residents, aged 18+ years, having contact (face‐to‐face/video/phone) with a healthcare worker in the past month (≥ 10 min). During metric validation, obtaining varied perspectives via a diverse sample is crucial [30] so wide‐ranging recruitment avenues were used. The study was advertised via; social media (Twitter, LinkedIn), third sector organisations, the University Research Participation Scheme, and posters in local community centres in Bristol. Thirty‐five third sector organisations were approached, and five shared advertisements (organisations focussed on mental health, physical health, long‐term conditions, and community groups). All interviewees were offered £15 reimbursement. To ensure participants were genuine, respondent demographics were reviewed. Respondents with non‐UK postcodes or incomes not in pounds sterling were excluded. Interview invites via Calendly captured geographic region further identifying non‐UK respondents. Common practice to achieve data saturation in cognitive interviews for metric validation ranges from 5 to 15 interviews [24, 30, 31, 32, 33, 34]. A sample of 15–20 participants was sought.

#### 2.2.3 Measures

Mandatory recruitment pre‐forms included: participant information, consent, demographics (based on UK census) and questions about the healthcare appointment. During interview, participants completed the 21 candidate items from initial CHIA development [16] before reviewing the draft 6‐item CHIA [25]. Both scales used a 7‐point Likert scale (1 = *Not at all*, 7 = *Completely*) with a ‘Not Applicable’ option (Supporting Information: Appendix 2).

#### 2.2.4 Procedure

All cognitive interviews were via video on Microsoft Teams. Interviews were recorded and transcribed verbatim then reviewed by the researcher or a final year undergraduate psychology student research apprentice (RA) for accuracy. There were two RAs (1 male, 1 female in their 20 s), both with research experience.

Pre‐interview all interviewees were reminded of key details from the consent form and briefed on the definition of compassion (verbally and using written resources) consistent with the definition from the previous Delphi [16]. All interviews followed the same topic guide (Supporting Information: Appendix 3) and were conducted by the same researcher (SA). Interviews followed the Three‐Step Test Interview method [27]:

*Step1:* Participants were introduced to think‐aloud and given a training task then completed the 21 CHIA candidate items [16] on their recent healthcare appointment whilst thinking‐aloud.

*Step2:* Probes were used to clarify or understand participants' stream‐of‐thought. To minimise confirmation bias, all probes occurred after participants finished their thought stream per item [30].

*Step3:* Participants were shown the reduced 6‐item draft CHIA and questioned on their perceptions of the questionnaire and items retained. The six‐item draft questionnaire was introduced in step three to prevent response bias. Probes included: (1) impressions of the draft six‐item metric (relative to compassionate care), (2) identifying whether the six items retained were most important, (3) if they would exclude or change items (i.e., add items from the original 21 CHIA candidate items) and (4) perceptions of domains.

#### 2.2.5 Analysis

A modified version of Peterson et al.'s [30] analysis model was followed. Dual coders were used to manage bias (SA and 2 RAs). Coders met frequently to review; any disagreements were discussed and resolved. The first five interviews were independently coded by all coders then discussed to ensure consistency; the remaining interviews were independently coded by the researcher and one RA then coders met to reach consensus. Interviewee responses were coded to identify congruence with existing item domains created during previously published content validity phases and finalised in the Delphi (Chatburn et al. [16]): Understanding, Attention, Communication, Action, Emotional Sensitivity, and Connection. Additionally, coders reviewed responses for question understanding and usability including applicability to their health appointment. Inter‐rater reliability was calculated using Fleiss Kappa and showed excellent reliability across raters for all areas, *κ* = 0.99. A spreadsheet recorded congruence, understanding and usability of each item from the 21 CHIA candidate items per participant, with supporting quotes. Additional columns summarised inconsistencies, questions not applicable to appointment types, item suggestions for inclusion/exclusion, additional suggestions, impressions of the questionnaire and title.

Cognitive interview data were collectively synthesised for item reduction decisions. This included summaries of: (1) item inconsistencies in congruence, understanding or usability, (2) interviewee‐suggested item inclusion/exclusion data (highlighting items with higher/lower face validity) (3) emerging demographic or appointment factors and (4) general feedback.

### 2.3 Results

#### 2.3.1 Sample

87 volunteers met inclusion criteria. Aligned with requirements for diverse perspectives [30] the 87 volunteers were screened on demographic and appointment information and participants were purposively selected with varying backgrounds for interview. To interview participants soon after their health appointment (to protect data quality), selection was done in a rolling fashion. Participants were interviewed in stages and new volunteers screened against demographics of those already interviewed. 28 volunteers were offered an interview, of which four did not respond and three did not attend resulting in a sample of 21. Interviewees were from across the UK (England, Wales, Scotland). An overview of key demographics is in Table 2 (full demographics in Supporting Information: Appendix 4). Interviews lasted 43–110 min and occurred between September and December 2024. All 21 interviewees completed all items providing either numerical or ‘Not Applicable’ ratings hence there was no missing data.

**Table 2:** Cognitive interviews key demographics summary.

	Interviewed *N* (%)	Recruited %		Interviewed *N* (%)	Recruited %
**Sex**			**Ethnicity**		
Male	5 (24%)	28%	Asian/Asian British	6 (29%)	18%
Female	16 (76%)	70%	Black/African/Caribbean/Black British	4 (19%)	10%
Other	0 (0%)	1%	White	9 (43%)	59%
Prefer not to say	0 (0%)	1%	Mixed/Multiple Ethnic Groups	1 (5%)	8%
*Gender different from sex assigned at birth*	2 (10%)	11%	Other Ethnic Group	1 (5%)	5%
**Age**			**Religion**		
18–24	7 (33%)	21%	No religion	7 (33%)	41%
25–34	5 (24%)	31%	Christian	7 (33%)	42%
35–44	2 (10%)	18%	Hindu	1 (5%)	5%
45–54	3 (14%)	13%	Jewish	1 (5%)	1%
55–64	3 (14%)	9%	Muslim	4 (19%)	8%
65+	1 (5%)	8%	Sikh	0 (0%)	1%
			Any other religion	1 (5%)	2%
**Sexual orientation**			**Disability**		
Straight or Heterosexual	15 (71%)	73%	No disability	12 (48%)	34%
Gay or Lesbian	1 (5%)	6%	Learning disability	1 (4%)	7%
Bisexual	3 (14%)	14%	Mobility impairment	4 (16%)	15%
Pansexual	0 (0%)	1%	Hearing loss	1 (4%)	2%
Asexual	1 (5%)	3%	Visual impairment	0 (0%)	2%
Other sexual orientation	1 (5%)	2%	Other disability	7 (28%)	39%
			Neurodiversity	4 (19%)	18%
**Education**			**Type of appointment**		
No qualifications	0 (0%)	1%	Mental health	5 (24%)	32%
GCSEs or equiv.	1 (5%)	3%	Physical health	11 (52%)	57%
AS, A Level or equiv.	3 (14%)	11%	Administrative issue	0 (0%)	1%
NVQ or equiv.	2 (10%)	5%	Other	5 (24%)	9%
Degree or equiv.	7 (33%)	46%	*Interviewees describing long‐term or chronic physical health conditions*	7 (33%)	
Postgraduate degree	8 (38%)	33%			

#### 2.3.2 Item Feedback

Cognitive interviews consistently highlighted 21 items as too many and potentially repetitive, but six items as too few and insufficient for good face validity (all items in Table 3). On the 6‐item draft metric only one item was unanimously retained by interviewees (Item 7) (Supporting Information: Appendix 2 shows 6‐item scale). The other five items were suggested for removal with varying consensus. Of the remaining 15 items from the 21 candidate items all were suggested for retention to varying degrees by interviewees: five by more than a quarter of the sample (Items 1, 2, 3, 13, 21).

**Table 3:** Analyses of the 21 CHIA candidate items pre item removal (Study 2 in Figure 2).

	% NA's	Inter‐item correlations	IDI	Factor loading	Item fit (*p*‐Value)	Local dependency
1. The things that matter most to me were understood.	3.54%	0.76–0.88	5.16	0.92	0.056	
2. I was taken seriously.	0.96%	0.77–0.88	6.83	0.93	0.079	
3. The staff member showed me they had listened to me (e.g., by what they said or what they did).	1.93%	0.78–0.86	6.42	0.92	0.042	0.301 (with Q8)
4. The staff member made time for me.	0.64%	0.71–0.89	5.05	0.9	0.080	
5. The staff member explained clearly what was happening to me and what would happen next.	6.11%	0.75–0.85	4.21	0.89	0.057	0.283 (with Q7)
6. I was given clear answers to my questions.	6.75%	0.78–0.86	4.85	0.91	0.059	
7. The staff member explained things to me in a way that I understood.	2.25%	0.70–0.81	3.74	0.84	0.039	
8. Information was shared in a sensitive way (e.g., test results, treatment plans).	22.19%	0.73–0.83	4.64	0.89	0.015	0.221 (with Q1)
9. The staff member acted in my best interests.	3.22%	0.76–0.88	6.26	0.93	0.013	0.222 (with Q16)
10. The staff member made me feel safe.	6.75%	0.74–0.87	5.44	0.92	0.000	
11. I was involved as far as possible in all decisions about my care.	6.11%	0.69–0.82	3.71	0.85	0.036	
12. The staff member did what they said they would do.	9.65%	0.71–0.83	3.57	0.86	0.064	0.292 (with Q4)
13. The staff member showed that they genuinely cared.	0.64%	0.76–0.90	6.79	0.94	0.024	
14. The staff member showed that they wanted to help me.	0.64%	0.79–0.90	7.43	0.94	0.078	
15. The staff member showed that they could see things from my perspective.	4.82%	0.74–0.88	5.18	0.91	0.000	
16. The staff member was comfortable discussing sensitive issues with me.	15.43%	0.69–0.84	4.35	0.89	0.066	0.217 (with Q11)
17. The staff member allowed me to be honest about my feelings.	5.79%	0.74–0.84	4.33	0.88	0.051	0.220 (with Q9)
18. I trusted the staff member.	0.64%	0.76–0.84	5.65	0.9	0.000	
19. The staff member treated me with respect.	0.64%	0.74–0.88	6.23	0.92	0.064	0.355 (with Q20)
20. I was treated as a fellow human being.	1.29%	0.75–0.88	5.83	0.91	0.073	0.214 (with Q1)
21. The staff member treated me with kindness.	0.64%	0.73–0.89	6.24	0.91	0.073	0.287 (with Q3)

*Note:* Greyed out rows are items subsequently removed from the metric based on cognitive interview and survey data.

Abbreviations: CHIA, Compassionate Healthcare In Action; IDI, item discrimination index; NA, not applicable.

#### 2.3.3 Item Use Factors

Healthcare appointment specific factors affected 15 items and related to appointment types that made rating difficult or resulted in ‘Not applicable’ answers. Potential ceiling effects were observed in four items, a potential central tendency effect for one item and difficulties with dual wording of another. Some differences in how participants interpreted item wording was observed but was not consistent. Explicit positive feedback on items was collated.

#### 2.3.4 Population Factors

Questions were raised about the metric's use in different populations (e.g., mental vs. physical health, chronically ill populations or for carers/parents) which will be important for future research.

## 3 Study 2: Scale Development

### 3.1 Research Questions

a. What items should be retained/removed to optimise the integrity of the metric?
b. What factor structure best achieves internally consistent and stable domains?

### 3.2 Materials and Method

#### 3.2.1 Design

Study 2 used a cross‐sectional survey design.

#### 3.2.2 Recruitment

Inclusion criteria were UK resident, aged 18+ years, having contact (face‐to‐face/video/phone) with a healthcare worker in the past month. The study was advertised via; social media (Twitter), third sector organisations, and the University Research Participation Scheme. Third sector charities focussed on mental health, long‐term conditions, neurological problems, neurodevelopmental difficulties and cancer. A Captcha test at the beginning of recruitment forms reduced non‐genuine participants. Participants were not paid but could opt‐in to win a gift voucher.

#### 3.2.3 Measures

Participants completed a demographics questionnaire and questions about their recent healthcare appointment. All participants completed the original 21 CHIA candidate items [16].

#### 3.2.4 Procedure

Participants completed online questionnaires (although could request paper‐based). All participants were provided with a study information sheet and gave informed consent. They were asked to recall a specific contact with a healthcare worker within the last month and asked questions about that appointment. Participants completed the CHIA items in reference to that appointment then were provided a debrief.

#### 3.2.5 Analysis

The CHIA uses a Likert scale and produces polytomous data so the Graded Response Model (GRM) within Item Response Theory was used [35]. Analysis was run in RStudio [36]. There was no missing survey data, however, the metric had a ‘Not Applicable’ option to understand the universality of items. Consequently, the GRM analysis excluded ‘Not Applicable’ cases pairwise.

Inter‐item correlations were calculated using Spearman's correlation as data was non‐normal. Guidance on interpreting inter‐item correlations suggests a consistent lower cut‐off of 0.30 [24, 37] whereas upper cut‐off guidance varies between 0.90 [37, 38], 0.95 [39] or no upper threshold [24]. The Item Discrimination Index (IDI) was calculated and interpreted using the Baker and Seock‐Ho [40] cut‐off of 1.35 (high) and 1.70 (very high). The exploratory factor analysis (EFA) was run in the GRM using Minimum Residual extraction (as normality was violated) and direct oblimin (oblique) rotation. Scale internal consistency was calculated using Cronbach's alpha, interpretation used a cut‐off of 0.80 as good [37]. Model fit was interpreted using a good fit cut‐off of ≤ 0.05 for root mean square error of approximation (RMSEA) and standardized root mean square residual (SRMSR), and ≤ 0.08 (RMSEA) and ≤ 0.010 (SRMSR) as acceptable fit [35]. For Comparative Fit Index (CFI) and Tucker‐Lewis Index (TLI) good fit was ≥ 0.95 and acceptable fit was ≥ 0.90 [41].

Item fit analysis in RStudio [36] does not allow removal of cases pairwise, hence for item fit analysis ‘Not Applicable’ cases were removed listwise. Item fit statistics were interpreted using a cut‐off of RMSEA ≤ 0.05 and non‐significant item fit statistics [35]. Finally, local dependencies were interpreted using Yen's *Q*_3_ statistic [42] with a critical cut‐off of 0.2 for local dependency issues (0.3 indicating severe local dependency) [43].

#### 3.2.6 Review Panel

Collated quantitative (survey) and qualitative (cognitive interview) data were initially reviewed by core members of the research team (SA, RP and LM) bringing previous expertise in compassion, cognitive interviews and metric development/validation. Suggested changes were taken to a wider panel, which following Patient‐Reported Outcomes Measurement Information System (PROMIS) [15] guidance involved additional content and measurement experts alongside patients. Alongside the core team, a further compassion expert, experts in cognitive interviews, statistical analysis, metric validation, the lived experience consultant, and RAs were included (*n* = 8). The panel met following analysis to discuss insights and inform item reduction decisions. Results from the earlier Delphi [16] were respected; recognition of item domains and high ratings of relevance. Reviewers considered both quantitative (statistical analyses) and qualitative (cognitive interview) data from the present study equally.

#### 3.2.7 Re‐Analyses

Following panel discussion, statistical analyses were re‐run removing excluded items individually to assess impact on statistical results. The final CHIA was restructured so item order reflected the user journey in a health appointment based on cognitive interview feedback. The ‘Not Applicable’ option was removed, administration and scoring guidance added (individual items are summed to compute a scale score).

### 3.3 Results

#### 3.3.1 Sample

311 participants completed the survey between October 2020–2021. Two participants opted for paper‐based; all others completed the survey online. The sample was predominantly female (71% female, 27% male, 1% other, 0.3% prefer not to say) and identified as White ethnicity (2% Asian or Asian British, 1% Black or Black British, 3% Mixed, 95% White and 0.3% Other Ethnicity). Participant ages varied with 25% aged 18–30, 36% aged 31‐60 and 38% aged 61–91. Participants completed the CHIA regarding varied appointment contexts and health concerns. For full demographics, appointment factors and health concerns see Supporting Information: Appendix 5.

#### 3.3.2 Analyses

There was no missing data, all participants provided numerical or ‘Not Applicable’ ratings for each item. All items had some ‘Not Applicable’ data (Table 3). Nine items had ‘Not Applicable’ below 2%, four below 5% and six below 10%. Two items had high ‘Not Applicable’ percentages of 15.43% (Item 16) and 22.19% (Item 8). Inter‐item correlations ranged from 0.69 to 0.91 with 0.69 and 0.91 only affecting 1 pairing each. This suggests all items have high common variance likely measuring the same construct.

All IDI scores exceeded the ‘Very High’ cut‐off of 1.70 suggesting every item clearly differentiates between high and low scores (Table 3). Examination of step calculations showed threshold parameters decreased across the scale suggesting individuals experiencing compassionate care levels as lower are more likely to choose lower response categories. However, individuals experiencing compassionate care as around the mean are more likely to opt for the highest extreme category or its adjacent response. This might suggest the scale is less sensitive to individuals experiencing compassionate care levels as high.

The EFA on the 21 candidate items yielded a single factor solution explaining 0.897 of the variance, with factor loadings > 0.8. The SRMSR (0.04), TLI (0.98) and CFI (0.98) demonstrated good model fit exceeding relevant cut‐offs whereas RMSEA (0.11) did not meet acceptable or good fit. Cronbach's alpha was 0.99, this could suggest good internal consistency. Equally, such a high alpha could imply item redundancy.

Item fit analyses (*n* = 171) identified 13 items with item fit concerns. Larger samples increase the likelihood of significant item fit scores [35] so 13 items with concerns is unsurprising. However, only 10 items were flagged for potential local dependency (Table 3).

#### 3.3.3 Review Panel

Consensus was reached on removal of six items (items 5, 8, 12, 16, 17, 20) which demonstrated statistical concerns (local dependency issues, ‘Not Applicable’ > 5%; (Table 3)) and poor face validity from cognitive interviews. Three further items were debated (items 9, 10 and 14). Item 9 was felt crucial to the conceptualisation of compassionate care and measuring observable behaviours. Also, its local dependency issue was with Item 16 which was being removed. Hence, researchers retained Item 9. Item 10 was contentious due to its varied interpretation by interviewees. On balance researchers agreed the item was not essential and risked introducing error so removed it. Item 14 was debated due to the higher inter‐item correlation with Item 13 (0.91). Item 14 was less popular than Item 13 with interviewees but nevertheless popular, statistically robust and conceptually relevant. Additional analysis was needed to understand the ramifications of removing Item 14.

#### 3.3.4 Final Metric Analysis (14‐Items)

Inter‐item correlations ranged from 0.71 to 0.91 with 0.91 only affecting 1 item pairing. All IDI scores exceeded the ‘Very High’ cut‐off (1.70) suggesting items clearly differentiate. EFA yielded a single factor solution explaining 0.914 of the variance, with factor loadings > 0.80. Model fit statistics all met either good (SRMSR = 0.02, TLI = 0.99, CFI = 0.99) or acceptable thresholds (RMSEA = 0.08). Cronbach's alpha remained 0.99 suggesting good internal consistency and/or potential item redundancy. Item reduction meant listwise deletion for item fit analysis yielded a sample of 254. Consequently, given larger samples increase significant item fit scores [35] item fit difficulties were observed for all items. However, local dependency issues reduced to six items. Item results are reported in Table 4.

**Table 4:** Analyses of the 14‐item CHIA post item removal (Study 2 in Figure 2).

	% NA's	Inter‐item correlations	IDI	Factor loading	Item fit (p value)	Local dependency
1. The things that matter most to me were understood.	3.54%	0.76–0.87	5.50	0.92	0.000	
2. I was taken seriously.	0.96%	0.77–0.88	7.15	0.93	0.013	0.228 (with Q4)
3. The staff member showed me they had listened to me (e.g., by what they said or what they did).	1.93%	0.79–0.86	6.71	0.93	0.017	0.279 (with Q13)
4. The staff member made time for me.	0.64%	0.73–0.89	5.35	0.91	0.000	
6. I was given clear answers to my questions.	6.75%	0.78–0.86	5.12	0.91	0.027	
7. The staff member explained things to me in a way that I understood.	2.25%	0.70–0.81	3.72	0.84	0.057	
9. The staff member acted in my best interests.	3.22%	0.76–0.88	6.75	0.93	0.042	0.340 (with Q18)
11. I was involved as far as possible in all decisions about my care.	6.11%	0.70–0.82	3.83	0.85	0.031	
13. The staff member showed that they genuinely cared.	0.64%	0.76–0.90	7.11	0.94	0.041	0.225 (with Q2)
14. The staff member showed that they wanted to help me.	0.64%	0.79–0.90	7.85	0.94	0.055	0.212 (with Q3)
15. The staff member showed that they could see things from my perspective.	4.82%	0.74–0.88	5.59	0.92	0.029	
18. I trusted the staff member.	0.64%	0.76–0.84	5.75	0.9	0.046	
19. The staff member treated me with respect.	0.64%	0.74–0.87	5.97	0.92	0.000	
21. The staff member treated me with kindness.	0.64%	0.73–0.89	6.08	0.91	0.046	0.281 (with Q3)

*Note:* The item numbering from the original 21 CHIA candidate items is retained in this table to make comparison between Table 3 and Table 4 easier.

Abbreviations: CHIA, Compassionate Healthcare In Action; IDI, item discrimination index; NA, not applicable.

#### 3.3.5 Additional Analysis

An analysis was run excluding Item 14 to understand if exclusion offered statistical benefits (Supporting Information: Appendix 6). The inter‐item correlations, IDI and factor loadings remained largely unchanged. Existing local dependency issues predominantly increased, Cronbach's alpha decreased (0.98) as did model fit statistics (RMSEA = 0.09, SRMSR = 0.03, TLI = 0.99, CFI = 0.99). Notably RMSEA no longer met acceptable fit and the proportion of variance explained reduced to 0.900. Consequently, Item 14 was retained as interviews indicated high face validity and removing it negatively impacted the metric's statistical robustness without improving item redundancy concerns.

#### 3.3.6 Item Reduction Decisions

Overall, researchers removed seven items (items 5, 8, 10, 12, 16, 17 and 20). Items were chosen due to a combination of ‘Not applicable’ levels, usability issues in interviews, local dependency issues and item face validity (recommendations for retention/removal). This resulted in a 14‐item metric across six domains (Supporting Information: Appendix 7) which was consistent with interview feedback that a scale between 21 and 6 items was desirable, and all themes were valuable.

## 4 Study 3: Scale Evaluation

### 4.1 Research Questions

a. Does the CHIA have robust dimensionality?
b. Is the CHIA reliable (internal consistency and test‐retest)?
c. Does the CHIA demonstrate strong validity (convergent and discriminant)?

### 4.2 Materials and Methods

#### 4.2.1 Design

Study 3 used a cross‐sectional survey design.

#### 4.2.2 Recruitment

Inclusion criteria were UK resident, aged 18+ years, having contact (face‐to‐face/video/phone) with a healthcare worker in the past month (≥ 5 min). The appointment window shortened to allow for GP appointments. The study was advertised via social media (LinkedIn, Instagram), third sector organisations, the University Research Participation Scheme, and posters in local community centres in Bristol, Bath and London. Third sector charities focussed on mental health, physical health, long‐term conditions, and target groups (e.g., men, older adults, ethnic minorities). A Captcha test at the beginning of recruitment forms reduced non‐genuine participants. Participants were not paid but could opt‐in to win a gift voucher. Boateng et al.'s [24] recommended sample size is 200–300 or 10–20 participants per item. Therefore, the target sample was 200–280.

#### 4.2.3 Measures

Participants completed the same demographics questionnaire and healthcare appointment questions used during cognitive interview. All participants completed the 14‐item CHIA (7‐point Likert scale (1 = *Not at all*, 7 = *Completely*)). To assess convergent and discriminant validity additional metrics were used (Table 5).

**Table 5:** Metrics used to assess convergent and discriminant validity.

Measure	Description
*Sinclair Compassion Questionnaire (SCQ)* [21]	The SCQ is a 15‐item measure assessing compassionate healthcare on a 5‐point Likert scale (1= Strongly disagree, 5 = Strongly agree). It has excellent internal consistency (*α* = 0.96), test‐ retest reliability (intraclass correlation coefficients: 0.74–0.89), and strong convergent validity with SCCCS (*r* =0.75, *p* < 0.001).
*Schwartz Center Compassionate Care Scale (SCCCS)* [18]	The SCCCS is a 12‐item measure assessing perceptions of compassionate care on a 10‐point Likert scale (1 = Not at all, 10 = Very successfully). It has excellent internal consistency (*α* = 0.98), test‐retest reliability (0.90), and convergent validity with the CARE measure (*p* < 0.001).
*Consultation And Relational Empathy (CARE)* [19]	The CARE is a 10‐item measure assessing clinician empathy on a 5‐point Likert scale (1 = Poor, 5 = Excellent). This measure has excellent internal consistency (*α* = 0.92) and good convergent validity with another empathy measure (Reynolds Empathy Scale; *r* = 0.85).
*Five‐item tool to measure patient assessment of clinician compassion (TMPACC)* [20]	The TMPACC is a 5‐item measure assessing perceptions of clinician compassion on a 4‐point Likert scale (1 = Never, 4 = Always). It has excellent internal consistency (*α* = 0.94) and positively correlated with overall patient satisfaction (*r* = 0.66).
*HowRwe* [44]	This assesses generic patient experience and is well‐validated. Participants rate 4‐items from Excellent to Poor, with lower total scores indicating a better patient experience.
*Overall satisfaction rating (OSR)*	Participants rate how satisfied they are with the overall healthcare interaction on a 7‐point Likert scale (1= Not at all, 7= Completely).
*HowRu* [45]	This well‐validated measure assesses generic health outcomes across four areas (mood, pain, restrictions, requiring help from others). Each area is rated from None to Extreme. Lower total scores indicate better well‐being.
*Generalized Anxiety Disorder 7‐item (GAD‐7)* [46]	The GAD‐7 is a 7‐item measure assessing generalised anxiety on a 4‐point Likert scale (1 = Not at all, 4 = Nearly every day). It has good reliability (*α* = 0.89) and good convergent validity with other anxiety measures (Penn State Worry Questionnaire‐Abbreviated; *r* = 0.70).

#### 4.2.4 Procedure

Participants completed an online questionnaire previously piloted by three individuals with experience of accessing physical health services (paper versions were available). After providing informed consent, participants were asked to complete questionnaires regarding a specific contact with a healthcare worker within the last month. Questionnaires were randomised in QuestionPro to reduce order bias. Participants were debriefed and given the option to re‐complete the survey a week later (for test‐retest).

#### 4.2.5 Analysis

Data was analysed in RStudio [36]. Missing values were examined visually and via Littles MCAR. Potential outliers were examined using stem‐and‐leaf plots. Assumptions of normality were investigated using histograms, Q‐Q plots and Shapiro–Wilk tests. Linearity was investigated using average Spearman correlations and homogeneity of variance was assessed using Levene's test. Given the non‐normality of data a Spearman's correlation was used to assess multicollinearity. The same correlation thresholds as the EFA were used for consistency. Confirmatory factor analysis (CFA) was conducted using the Weighted Least Squares Mean and Variance Adjusted estimator in the lavaan package in R [47] to account for non‐normality and heterogeneity. The model fit was assessed using multiple model fit indices as in the EFA.

Internal consistency was assessed using Cronbach's alpha and Mcdonald's omega, with a cut‐off of 0.80 as good [37]. Test‐retest reliability was calculated using two‐way mixed‐effects Intraclass Correlation Coefficients (ICC) for absolute agreement (ICC(2,1)) and consistency (ICC(3,1)) between total CHIA at initial survey and a week later [48]. Cut‐offs to aid interpretation were: <0.50 poor, 0.50‐.75 moderate, 0.75‐.90 good, >0.90 excellent [49]. Convergent validity was calculated using Spearman correlation coefficients between the CHIA and other compassionate care PREMs (SCQ, SCCCS, CARE, TMPACC), and patient satisfaction measures (OSR, HowRwe). Discriminant validity was assessed using Spearman correlation coefficients between the CHIA and wellbeing measures (HowRu, GAD‐7). No universal cut‐off for convergent validity is recognised, however cut‐offs of < 0.3 (weak), 0.3‐0.6 (moderate) and > 0.6 (strong) are frequently used [50]. These cut‐offs were used in the present study. For discriminant validity accepted practice is as near 0 as possible.

### 4.3 Results

#### 4.3.1 Sample

301 participants were included in the study between May and July 2025. Participants had varied demographics and completed the CHIA regarding varied appointment contexts and health concerns. Participants were from across the UK (England, Wales, Scotland, Ireland). For full demographics, appointment factors and health concerns see Supporting Informatiob: Appendix 8. A brief summary is in Table 6.

**Table 6:** Survey demographics summary.

	*n*	%		*n*	%
**Sex**			**Ethnicity**		
Male	61	20.3%	Asian/Asian British	34	11.3%
Female	231	76.7%	Black/African/Caribbean/Black British	18	6%
Other	1	0.3%	White	225	74.8%
Prefer not to say	4	1.3%	Mixed/Multiple Ethnic Groups	12	4%
Not available	4	1.3%	Other Ethnic Group	9	3%
*Gender different from sex assigned at birth*	16	5.3%	Not available	3	1%
**Age**			**Sexual orientation**		
18–24	111	36.9%	Straight or Heterosexual	243	80.7%
25–34	47	15.6%	Gay or Lesbian	8	2.7%
35–44	30	9.9%	Bisexual	30	10%
45–54	50	16.6%	Pansexual	5	1.7%
55–64	37	12.3%	Asexual	8	2.7%
65+	23	7.6%	Other sexual orientation	4	1.3%
Not available	3	1%	Not available	3	1%
**Religion**			**Disability**		
No religion	154	51.2%	No disability	218	64.1%
Buddhist	3	1%	Learning disability	12	3.5%
Christian	115	38.2%	Mobility impairment	14	4.1%
Hindu	8	2.7%	Hearing loss	9	2.6%
Jewish	1	0.3%	Visual impairment	9	2.6%
Muslim	5	1.7%	ADHD	14	4.1%
Sikh	3	1%	Autism	40	11.8%
Any other religion	8	2.7%	Dyslexia	3	0.88%
Not available	4	1.3%	Tourettes Syndrome	1	0.29%
			Suspected/Undiagnosed	1	0.29%
			Other Neurodivergence	2	0.59%
			Chronic illness	62	20.6%
			Unclear	13	0.38%
			Caregiver (someone with chronic illness)	18	6%
**Education**			**Type of appointment**		
No qualifications	5	1.7%	Mental health	56	18.6%
GCSEs or equiv.	19	6.3%	Physical health	199	66.1%
AS, A Level or equiv.	38	12.6%	Social Problem	1	0.3%
NVQ or equiv.	7	2.3%	Administrative issue	5	1.7%
Degree or equiv.	127	42.2%	Other	40	13.3%
Postgraduate degree	106	33.9%			
Not available	3	1%			

#### 4.3.2 Preliminary Analyses

The SCCCS had missing data so pairwise deletion was used for analyses including this scale. The Shapiro–Wilk test indicated the CHIA was non‐normal (*W* = 0.88, *p* < 0.001) and Levene's test rejected the assumption of equal variance between CHIA items (*F* = 3.02, *p* < 0.001). The CHIA had strong linearity with average inter‐item correlation of 0.75 and no item correlations outside cut‐offs. Research suggests ceiling and floor effects are present if 15%–20% of individuals select the best or worst possible score [51]. Floor effects were absent but ceiling effects were between 29% and 54%. Table 7 shows descriptive statistics for CHIA items and appendix 9 highlights ceiling effects for other compassionate care PREMs used.

**Table 7:** 14‐item CHIA descriptive statistics.

	Inter‐item correlations	Min	Max	*M*	SD	Skew	Kurtosis	Ceiling effects
1. I was taken seriously.	0.68–0.81	1	7	5.67	1.48	−1.07	0.40	39.5
2. The staff member showed me they had listened to me.	0.73–0.82	1	7	5.58	1.49	−0.91	−0.05	36.9
3. The staff member showed that they could see things from my perspective.	0.65–0.84	1	7	5.27	1.61	−0.82	−0.01	29.2
4. The things that matter most to me were understood.	0.65–0.84	1	7	5.25	1.72	−0.78	−0.35	32.2
5. The staff member made time for me.	0.68–0.83	1	7	5.57	1.57	−1.05	0.31	38.5
6. The staff member treated me with respect.	0.65–0.80	1	7	6.02	1.40	−1.64	2.19	53.5
7. The staff member showed that they wanted to help me.	0.74–0.87	1	7	5.69	1.55	−1.22	0.71	42.5
8. The staff member showed that they genuinely cared.	0.72–0.87	1	7	5.40	1.69	−0.96	0.07	35.6
9. The staff member explained things to me in a way that I understood.	0.70–0.82	1	7	5.74	1.52	−1.21	0.78	44.5
10. I was given clear answers to my questions.	0.65–0.82	1	7	5.39	1.71	−0.87	−0.25	37.2
11. I was involved as far as possible in all decisions about my care.	0.67–0.81	1	7	5.58	1.58	−1.06	0.38	39.5
12. The staff member acted in my best interests.	0.73–0.82	1	7	5.64	1.60	−1.17	0.57	42.2
13. I trusted the staff member.	0.70–0.80	1	7	5.49	1.69	−1.03	0.18	39.5
14. The staff member treated me with kindness.	0.68–0.80	1	7	5.82	1.51	−1.43	1.50	46.5

Abbreviations: CHIA, Compassionate Healthcare In Action; SD, standard deviation.

#### 4.3.3 Dimensionality, Validation and Reliability

##### 4.3.3.1 Dimensionality

The CFA supported a single‐factor structure in the CHIA with standardised factor loadings from 0.86 to 0.96. There was adequate or good model fit for most indices (CFI = 0.92, TLI = 0.90, SRMSR = 0.03). However, the RMSEA (0.16, CI90% = 0.14‐0.17) indicated poor model fit (*χ* ^2^/*df* = 226/77 = 2.9), so standardised residuals were examined to establish if misfit was general or localised to specific items. This analysis showed misfit was generally present across multiple item pairings.

##### 4.3.3.2 Validity

The CHIA demonstrated strong convergent validity with other compassionate care PREMs and moderately/strongly correlated with satisfaction measures in expected directions (HowRwe is scored in the opposite direction hence the negative correlation). The CHIA demonstrated strong discriminant validity as evidenced by the weak correlation with mood and wellbeing measures. Table 8 shows correlation coefficients.

**Table 8:** Spearman correlations between the CHIA and other measures.

Concept	Name of measure	Correlation coefficients
Compassionate Care	CARE	0.79*
	SCQ	0.76*
	TMPACC	0.62*
	SCCCS	0.64*
Satisfaction	HowRwe	−0.56*
	OSR	0.79*
Mood and Wellbeing	HowRU	− 0.14*
	GAD‐7	−0.14*

Abbreviations: CARE, Consultation and Relational Empathy Scale; CHIA, Compassionate Healthcare In Action; SCQ, Sinclair Compassion Questionnaire; TMPACC, Tool to Measure Patient Assessment of Clinician Compassion; SCCCS, Schwartz Center Compassionate Care Scale; OSR, Observation Screening Response; GAD‐7, Generalized Anxiety Disorder 7‐item scale.

* Correlation is significant at the 0.05 level.

##### 4.3.3.3 Reliability

Reliability. The CHIA exhibited excellent internal consistency based on Cronbach's alpha (0.98) and McDonald's omega (0.98). Test‐retest reliability was analysed on 48 participants and demonstrated an estimated agreement of 0.76 (95%CI: 0.60–0.86), and estimated consistency of 0.77 (95%CI: 0.62–0.86). Both agreement and consistency estimates suggest good test‐retest reliability aligned with cut‐offs [49].

## 5 Discussion

Overall, this paper sought to complete development and evaluation of a new measure of Compassionate Healthcare in Action. The CHIA offers strong conceptualisation for measuring patient experiences of compassionate care and whilst inductively developed, aligns with existing models of both compassion (CFT [5]) and compassionate care (Dewar and Nolan [6]). It offers a valid and reliable way to measure patient experiences of compassionate care meeting concerns raised in the literature [3]. Furthermore, the CHIA addresses gaps in existing metrics by (a) focussing on action‐orientated components of compassion, (b) validating across a wider range of healthcare contexts, and (c) involving patients throughout development. The final 14‐item CHIA was derived from the 21 CHIA candidate items from earlier development stages, and results from a thorough metric design process [15, 16, 24].

Earlier focus on statistical data over qualitative insights led to premature item reductions that undermined face validity [25]. In this paper, valuing quantitative and qualitative data led to a statistically strong metric retaining items with high face validity. Analysis found items high in statistical concerns often overlapped with items low in face validity. Some items differed and balance was sought between statistical evidence and qualitative feedback. Similarly, participants' views that 6 items felt too few and 21 too many reinforced the need for caution and thoroughness in item reduction. This underlines Boateng et al.'s [24] argument for a robust development cycle in metric creation valuing both interview and survey data.

Study 3 of this paper reinforced findings that the 14‐item metric was stronger. However, there were some statistical discrepancies. The presence of local dependencies can increase model misfit by producing unexplained covariance (increasing residuals) [52, 53]. RMSEA calculates using residuals and doesn't account for correlations among item errors or clusters of related items. Thus, a poor RMSEA score was understandable given the high local dependence for 6‐items in the 14‐item CHIA. Considering the single‐factor solution, high local dependence was likely [54] but deemed acceptable by researchers to balance qualitative data and retain items high in face validity. Overall, the adequate fit of other model fit statistics suggests the CHIA successfully balances robust dimensionality with face validity.

Like RMSEA concerns, internal consistency of 0.98 whilst indicative of a reliable measure could also indicate item redundancy [55]. The suggestion that some items are statistically redundant is consistent with local dependency results. Yet, quantitative item redundancy does not necessarily undermine metrics' statistical robustness as it allows for concept coverage [56]. The scale development team balanced the need for statistical perfection, resolving it was better to accept a highly reliable scale with potential item redundancy to retain face validity. Additionally, the CHIA showed strong convergent validity with four PREMs of compassionate care [18, 19, 20, 21] demonstrating the CHIA is consistent with other metrics in the space. The CHIA also demonstrated good test‐retest reliability, showing it is consistent across time points.

The development and evaluation of the CHIA reinforces the importance of prioritising mixed‐methods designs. Overfocussing on one approach and proceeding too quickly can lead to poorer quality metrics that prioritise certain forms of validity or statistical parameters. Content and face validity place strong reliance on qualitative approaches. Conversely, dimensionality, reliability, convergent and discriminant validity use quantitative approaches [24]. Thus, both are valuable and result in metric development being an iterative, lengthy and multi‐faceted process, as all analytical and methodological factors must be considered throughout. The CHIA's development actively followed this multi‐faceted process to achieve robust outcomes whilst staying true to metric aims.

The thorough development process aligned with the aim of addressing gaps in existing metrics of compassionate care. Some existing metrics demonstrate difficulties in their conceptual clarity by assessing multiple concepts, and mostly or exclusively focussing on subjective items. The CHIA's development consciously focussed on one concept (patient experiences of compassionate care) using action‐orientated items throughout development. Researchers actively built content validity and fidelity to these aims in the original development paper during informant interviews, literature review and Delphi. The present paper continued this by ensuring cognitive interviewees kept the target concept and behavioural focus in mind. Likewise, the review panel returned to the inductively developed definition and behavioural focus throughout decision‐making.

To ensure the CHIA's development was thorough and generalisable researchers focussed on including varied healthcare populations. Existing metrics were validated on specific healthcare populations/professionals and all six were not validated for mental health populations potentially leading to context specific validity. 24% of those sampled in the present paper contributed from a mental health perspective, directly addressing this gap. Physical healthcare participants spanned a variety of contexts and professionals (see Supporting Information: S1).

Additionally, only two existing metrics report qualitatively involving patients in development. A lack of patient involvement can undermine metrics' content and face validity, adversely impacting their acceptance and usability [13, 14]. Consequently, the CHIA actively had PPI in every development stage to enable consultation on process and decisions. Development and validation maximised sampling for each phase facilitating diversity of input and actively valuing qualitative feedback from patients throughout.

Overall, the CHIA's development adhered to Boateng et al.'s [24] thorough development cycle whilst also actively seeking to build on existing literature and address gaps in current questionnaire provision. It has striven to build a metric with conceptual clarity, an action‐orientated focus, involved patients throughout development and evaluated with varied healthcare populations. The CHIA offers a strong alternative to assess patient experiences of compassionate care.

### 5.1 Limitations and Strengths

The present study demonstrated four main methodological limitations. First, diversity of samples, whilst good, could have been improved. Although there was diversity in the samples representing multiple ethnicities, religions, genders, sexualities, (dis)abilities, education and employment statuses, and representing multiple healthcare contexts (both mental and physical), the representation differed between recruitment, interview and survey. The rolling recruitment of interviews impacted diversity as earlier volunteers were more homogenous than later volunteers. Both surveys had majority white and female participants and there was a bias towards younger more educated responders. This may have shaped participants' item interpretation reflecting age and educational differences in language use. This could influence the generalisability of results and future research will have to confirm the metric's validity in more balanced samples.

Second, whilst cognitive interviews enable rich qualitative data and are recommended for establishing face validity [24, 26, 57], there are limitations to this method. Notably, differences in participants' language use, possibly reflecting English being a second language for some and/or variation in education, may have meant more articulate participants responses were weighted more strongly [58]. Additionally, interviews were completed in an artificial setting, not immediately post‐appointment which could have affected responses.

Third, the panel discussion included eight members but could have involved a larger panel of experts to provide a wider range of perspectives. However, the panel was sufficient to offer insight in both PROMIS identified expert areas and patient involvement aspects [15].

Finally, the metric demonstrated high ceiling effects. This could suggest the scale needs shortening or reflect sampling limitations (participants volunteering following experiences of perceived extremes in compassionate care). However, ceiling effects are common in PROMs generally [59] and are observed in other compassionate care scales [18, 19]. Notably, in the present study ceiling effects occurred in convergent validity scales too (Supporting Information: Appendix 9). Thus, whilst the ceiling effects suggest some limitations in differentiation, they are not anomalous with existing research. Furthermore, IDI scores during the EFA demonstrated strong differentiation despite this limitation.

Despite limitations, this study followed robust metric development methodology [24]. PPI throughout all studies enhanced the development processes and ensured patient voice was held strongly in mind. The studies had good volunteer rates for interviews and surveys which maximised analyses. Additionally, the diversity achieved in both interview and survey samples enhances the generalisability of validation results to the UK populace and across mental and physical healthcare contexts, although further research with patient subgroups is still recommended. Statistical analysis followed robust analytic principles to ensure insights could be interpreted accurately and inform decision making. Quantitative and qualitative data were both valued to ensure a robust and well‐rounded metric. The final CHIA items demonstrate strong content and face validity and the final metric evidences strong unidimensionality, reliability (internally and across time), convergent and discriminant validities.

### 5.2 Future Research

Whilst the CHIA was tested on large and diverse samples there was bias towards younger, educated, white and female respondents so future studies looking at more balanced samples would help to build a stronger picture of the CHIA's generalisability. During interviews researchers observed variation in how subgroups engaged with the questionnaire. Consequently, whilst the CHIA shows promise across multiple contexts and populations it would benefit from further validation with particular subgroups. It would be useful to understand how existing PREMs and the CHIA compare within specific subgroups. We suggest comparisons between different measures for mental health populations, chronically ill populations, acute settings and general physical health contexts as the nuances in patient experiences of compassionate care for each population may indicate differences in the suitability of each measure. Likewise, patients for whom English is a second language or who have differing language capabilities form distinct subgroups and future research could compare validity of the CHIA for these groups. Exploration of the CHIA's validity across men, women and other gender presentations may also be interesting, for example do different sexes or gender identities relate differently to the measure? Similarly, validation of metric completion within a healthcare setting (in situ) would improve the ecological validity of the CHIA.

To improve the CHIA's administration and interpretation it would be helpful to run studies into its scoring. Specifically, future research should consider the sum scale scoring to explore whether response categories or cut‐offs would be helpful and meaningful in scale interpretation. Likewise, the presence of ceiling effects, although consistent with existing measures, suggests there may be potential difficulties in discriminating responses. Future research could explore whether alternative response scale anchors or expanded response categories improve discrimination at the upper end of the scale.

### 5.3 Practical and Clinical Applications

The CHIA is designed to help understand patient experiences of compassionate care with healthcare providers. We hope it will prove a helpful feedback tool for services. Likewise, the CHIA could be used alongside questionnaires measuring barriers to compassionate care. This would support a rounded service evaluation and inform service improvement planning. Additionally, it could be used in development contexts to support healthcare professionals (particularly those in training) to reflect and build their compassionate care skills.

## 6 Conclusion

This paper aimed to address research and practice gaps in measurement of patient's experiences of compassionate care through the development of a novel measure of CHIA, with a focus on compassionate behaviours, application across physical and mental healthcare settings, and built using strong mixed‐method, PPI‐involving approaches. Researchers followed best practice metric development guidance by combining face validity analysis with statistical analysis to establish a well validated scale and items [24, 26]. The final 14‐item CHIA demonstrates robust single‐factor dimensionality across EFA and CFA alongside strong reliability (internal consistency, test‐retest) and validity (content, face, convergent and discriminant validities). It was created with patient involvement at every stage and could be useful for assessment of patient experiences of compassionate care in action in mental and physical healthcare.

## Author Contributions

**Samantha R. Allen:** conceptualisation, methodology, data curation, investigation, formal analysis, writing – original draft, writing – review and editing, validation. **Ellen Huish:** conceptualisation, methodology, investigation, data curation, formal analysis, writing – original draft, writing – review and editing, validation. **Roxanne M. Parslow:** conceptualisation, methodology, supervision, writing – original draft, writing – review and editing. **Elizabeth Marks:** conceptualisation, methodology, supervision, writing – original draft, writing – review and editing. **Lucy A. Clarkson:** conceptualisation, methodology, writing – review and editing. **Sarah Howard:** data curation, investigation, formal analysis, writing – original draft, validation. **Lucy Maddox:** conceptualisation, methodology, supervision, writing – original draft, writing – review and editing, validation.

## Conflicts of Interest

The authors declare no conflicts of interest.

## Supporting information


Supporting File

## Data Availability

The data that support the findings of this study are available on request from the corresponding author. The data are not publicly available due to privacy or ethical restrictions. The data that support the findings of this study are available on reasonable request from the corresponding author (with the exception of some participants who did not consent to this). The data are not publicly available due to ethical restrictions.
