# Supplementary material for: Further Development and Validation of a Measure of Compassionate Healthcare in Action
Source: Health Expect. 2026 Aug 3;29(4):e70796. doi: 10.1111/hex.70796 (PMC13430277; doi:10.1111/hex.70796)
Supplement: Supplementary file 1 — Supporting File [file HEX-29-e70796-s001.docx]

**SUPPLEMENTARY MATERIALS**

**CONTENTS**

|  | **Pg** |
| --- | --- |
| APPENDIX 1: Reporting Patient and Public Involvement in this Study Using GRIPP2 Short Form | 2-3 |
| APPENDIX 2: 21 candidate items & 6 draft items used in cognitive interviews - NOT VALIDATED FOR USE | 4-6 |
| APPENDIX 3: Interview Topic Guide | 7-15 |
| APPENDIX 4: Cognitive interviews full demographics | 16-17 |
| APPENDIX 5: Study 2 survey full demographics | 18 |
| APPENDIX 6: Item reduction additional analyses | 19 |
| APPENDIX 7: Final 14-item CHIA | 20-21 |
| APPENDIX 8: Study 3 survey full demographics | 22-23 |
| APPENDIX 9: Ceiling effects of the other compassionate care measures in study 3 | 24 |

**APPENDIX 1: Reporting Patient and Public Involvement in this Study Using GRIPP2 Short Form**

| **Section and topic** | **Item** | **Reported on page number** |
| --- | --- | --- |
| 1: Aim | The aim of PPI in the study was to ensure that the metric reflects the patient voice in this design and development in line with best practice. | 5 |
| 2: Methods | PPI ran throughout the previously reported item development for this measure. In the three studies reported in this current paper a PPI consultant was involved in reviewing methodology and materials, interviews and surveys for accessibility, giving advice and help with recruitment, contribution to the review panel analysis discussions during item reduction and review and contribution to this manuscript. The consultant was reimbursed for her time and is part of the paper authorship team. Methods throughout have sought to ensure that the voice of the people who will be completing this questionnaire form an important part of the data being considered in measure development. Larger samples were sought in both quantitative and qualitative parts of this research to maximise the diversity of patient voices. | 7 & 11 |
| 3: Study results | The results of the cognitive interviews and survey one were reviewed by our lived experience consultant to help support data interpretation, panel discussion and item reduction decisions. Their voice was key in decisions around which items to retain and they helped add weight to qualitative data from patients during interviews. Their comments on the final manuscript help to shape the paper. | 17 |
| 4: Discussion and conclusions | The PPI consultant has been involved in the questionnaire from start to finish shaping the metrics initial inception, methodological design and material development as well as interpretation and decision making subsequent to analysis. They have also been involved in the manuscript write-up and are co-authoring the paper. | 27-28 |
| 5: Reflections/critical perspectives | PPI input and guidance has been invaluable throughout the CHIA’s development and crucial to ensuring that the development processes allowed for sufficient areas for patient feedback through the Delphi, interviews and surveys. Patient and Public Involvement was critical to the design of this measure and has helped the research team to consider alternatives and make sense of qualitative feedback so that each development step is as meaningful as possible. This was particularly relevant during item retention discussions as purely quantitative data analysis may have led to further item reduction and PPI helped highlight the potential adverse impact to face validity and patient voice by not retaining more items. PPI consultancy input has been invaluable.  In addition, the methods throughout these studies sought to hold the perspectives of questionnaire respondents firmly in mind alongside statistical analyses, and the use of cognitive interviews and attempts to maximise diversity of the samples was also an important part of the process of questionnaire development which sought to prioritise patient perspectives.  A limitation to our PPI was that we had only one consultant through the process, although a benefit was that the same person has consulted on all studies. Ideally, we would have had more than one PPI consultant. | 28 |

**PPI = patient and public involvement*

**APPENDIX 2: 21 candidate items & 6 draft items used in cognitive interviews - NOT VALIDATED FOR USE IN SERVICES**

**21 candidate items used in cognitive interviews- NOT VALIDATED FOR USE IN SERVICES**

1. The things that matter most to me were understood.
2. I was taken seriously.
3. The staff member showed me they had listened to me (e.g., by what they said or what they did).
4. The staff member made time for me.
5. The staff member explained clearly what was happening to me and what would happen next.
6. I was given clear answers to my questions.
7. The staff member explained things to me in a way that I understood.
8. Information was shared in a sensitive way (e.g., test results, treatment plans).
9. The staff member acted in my best interests.
10. The staff member made me feel safe.
11. I was involved as far as possible in all decisions about my care.
12. The staff member did what they said they would do.
13. The staff member showed that they genuinely cared.
14. The staff member showed that they wanted to help me.
15. The staff member showed that they could see things from my perspective.
16. The staff member was comfortable discussing sensitive issues with me.
17. The staff member allowed me to be honest about my feelings.
18. I trusted the staff member.
19. The staff member treated me with respect.
20. I was treated as a fellow human being.
21. The staff member treated me with kindness.

**6 draft items used in cognitive interviews - NOT VALIDATED FOR USE IN SERVICES**

1. The staff member explained clearly what was happening to me and what would happen next.
2. The staff member explained things to me in a way that I understood.
3. I was involved as far as possible in all decisions about my care.
4. The staff member did what they said they would do.
5. The staff member allowed me to be honest about my feelings.
6. I was treated as a fellow human being.

**APPENDIX 3: Interview Topic Guide**


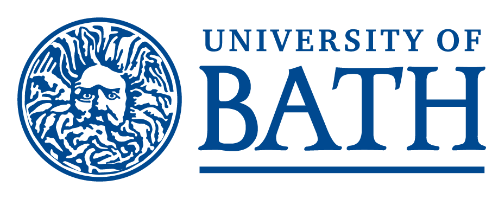


**Further Development and Validation of a Measure of Compassionate Healthcare In Action.**

**Cognitive Interviews Topic Guide.**

This topic guide shows the interview topics for the planned cognitive interviews. The interviewer may ask additional questions to clarify participants responses. In keeping with standard practice in qualitative research, these questions may be modified in the light of themes that emerge from earlier interviews.

# **Step 1: Introduction prior to interview**

The researcher will greet the participant and introduce themselves. Environmental distractions will be checked (If online – can they hear/see ok, are you somewhere quiet without risk of being overheard/interrupted; If f2f – is room set-up ok). The researcher will then reiterate the aims of the project and purpose of the interview. Participants will be reminded that the interview is recorded, they have a right to withdraw at any time (up till 31/03/2025) and transcripts will be anonymised.

## Script: project aims and interview purpose

- Help build a way of measuring the observable behaviours that professionals do that impact experiences of compassionate healthcare.
- Define compassion: (1) engagement – noticing and empathising when you or other people are upset and (2) action – proactively trying to get rid of or prevent that upset. (use visual aid in appendices).
  - Compassion is defined as: “a deep awareness of the suffering of another coupled with the wish to relieve it”
- Public helped create a 21-item questionnaire.
- We want to understand if the questionnaire works well is easy to understand and complete.
  - This means we would really like you to let us know if questions make sense and use the right language- for example, can you understand them, are they relevant to your healthcare experiences, can you select a response option that works well for you?
- This study is to help us understand which questions in our questionnaire are the most important.
- It went through statistical tests to make sure we found the best items which left us with 6 questions.
- Today have brought the 21-item and 6-item questionnaires as we want to check that we haven’t missed anything by going from 21 to 6 questions.

# **Step 2: Think-aloud**

The participant will complete a practice exercise then complete the questionnaire on their own whilst thinking aloud. Afterwards, the interviewer may ask some further clarification questions to probe participant responses.

## Script: task instructions and practice

- Going to give/send the 21-item questionnaire and ask you to read and complete each question as you would do normally, but whilst thinking-aloud.
- Think-aloud = read the question aloud and also say out loud any thoughts that come into you head when looking at the question. What healthcare are you thinking about, why did you pick the option that you did.
- Helps us to understand your immediate responses to the questions. Often when we speak we organise our thoughts so they sound more structured. The point of think-aloud is that we just want to hear your thoughts as they are happening before your brain structures them so it’s ok for your thoughts to jump around and change as you are saying them out loud.
- If there is any misunderstanding, go back through explanation
- **Practice**: visualize the place where you live and think about how many windows there are in that place. As you count up the windows, tell me what you are seeing and thinking about?
- Allow 5mins for task
- Prompt: “I can see that you are thinking about your answer, would you please speak your thoughts aloud?”

## Script: task and probes

- **Task**: want to know how you understand the questions, what they mean to you, your thoughts on the structure of the questionnaire, the response options. Give interviewee the 21-item questionnaire.
- Please can you go through each of the questions now in order and complete the questionnaire as you would do normally. Read each question aloud as we discussed then just speak out loud all your thoughts about that question as they happen. I may ask you some extra questions as we go through. Let me know when you are ready to move onto the next question in the list.

For example- tell me what healthcare experience you are thinking about, it can be either a positive or negative experience all experiences are helpful. Why are you selecting that response option. Don’t be afraid to say if a question doesn’t make sense or is not clear. That is what we want to understand.

- **Optional probes:**
- What is going through your mind?
- Please can you speak your thoughts aloud?
- What does that question make you think?
- How do you understand that question?
- Why did you pause, hesitate on that question?

# **Step 3: Semi-structured interview**

The participant will be given the 6-item questionnaire and asked structured questions to understand their impressions of this version compared to the 21-item version. They will also be given a sheet containing the 6 themes to review.

## Script: instructions

We wanted to make a shorter measure that is easier for people to complete. So, this shorter version was created by using statistics to identify the most useful questions statistically. I would like you to look at this questionnaire now and I have some questions for you about what you think of it.

## Script: specific questions

- Looking at the short questionnaire we have tried to make it shorter so it takes up less time for people but still measures compassion.
- What is your overall impression of the shorter questionnaire? Prompts: Are the questions important and relevant, do they capture compassionate care?
- Looking back at the long 21 item questionnaire…do you think any key items in the long one are missing from the short one? Why?
- The original 21 questions were found in our previous work to cover 6 themes, however, the 6-question version only includes 4 of those themes. If I show you the themes now (show appendix 1) do you think the 2 missed themes Understanding and Attention should be included?
  - If yes, which questions would you add back in? (show the questions to choose from) Why those ones?

# **Step 4: Debrief**

The participant will be debriefed using the debrief sheet.

# **Appendices**

There are 3 appendices:

1. Compassion definition
2. 21-item CCAM
3. 6-item CCAM
4. 6 themes previously identified





# **Questionnaire themes**

| **Understanding** | This theme includes items on being taken seriously, being understood, someone taking time to understand. |
| --- | --- |
| **Attention** | This theme includes items on listening, eye contact, body language, and time taken. |
| **Communication** | This theme includes items on clarity, explaining things, checking understanding, personalising information, and sharing personal information appropriately. |
| **Action** | This theme includes items on practical things and touch. |
| **Emotional sensitivity** | This theme includes items on showing upset, emotional care, kindness, tolerance of distress, and empathy. |
| **Connection** | This theme includes items on feeling cared for, trust, common humanity, dignity, respect, and being treated with kindness. |

| Theme | 21 candidate items | Reduced draft 6-item measure | |
| --- | --- | --- | --- |
| Understanding | - The things that matter most to me were understood. - I was taken seriously. | |  |
| Attention | - The staff member showed me they had listened to me (e.g., by what they said or what they did). - The staff member made time for me. | |  |
| Communication | - The staff member explained clearly what was happening to me and what would happen next. - The staff member explained things to me in a way that I understood. - I was given clear answers to my questions. - Information was shared in a sensitive way (e.g., test results, treatment plans). | | - The staff member explained clearly what was happening to me and what would happen next. - The staff member explained things to me in a way that I understood. |
| Action | - I was involved as far as possible in all decisions about my care. - The staff member did what they said they would do. - The staff member acted in my best interests. - The staff member made me feel safe. | | - I was involved as far as possible in all decisions about my care. - The staff member did what they said they would do. |
| Emotional sensitivity | - The staff member allowed me to be honest about my feelings. - The staff member showed that they genuinely cared. - The staff member showed that they wanted to help me. - The staff member showed that they could see things from my perspective. - The staff member was comfortable discussing sensitive issues with me. | | - The staff member allowed me to be honest about my feelings. |
| Connection | - I was treated as a fellow human being. - I trusted the staff member. - The staff member treated me with respect. - The staff member treated me with kindness. | | - I was treated as a fellow human being. |

**APPENDIX 4: Cognitive interviews full demographics**

**TABLE a.** Demographics of participants.

| **Sex** | **Ethnicity** | **Religion** |
| --- | --- | --- |
| Male 5  Female 16 | Asian/Asian British 6  Black/African/Caribbean/Black British 4  White 9  Mixed/Multiple Ethnic Groups 1  Other Ethnic Group 1 | No religion 7  Christian 7  Hindu 1  Jewish 1  Muslim 4  Any other religion 1 |
| **Gender different from sex assigned at birth** |  |  |
| Yes 2  No 19 |  |  |
| **Age** | **Sexual orientation** | **Current employment** |
| 18-24 7  25-34 5  35-44 2  45-54 3  55-64 3  65+ 1 | Straight or heterosexual 15  Gay or Lesbian 1  Bisexual 3  Asexual 1  Other sexual orientation 1 | Employee 12  Retired 1  Studying 4 Long-term sick or disabled 2  None of the above 2 |
| **Highest level of academic attainment** | **Disability** | **Household size** |
| GCSEs or equiv. 1  AS, A Level or equiv. 3  NVQ or equiv. 2  Degree or equiv. 7  Postgraduate degree 8 | No disability 12  Learning disability 1  Mobility impairment 4  Hearing loss 1  Other disability 7  Neurodiversity 4 | One 0  Two 5  Three 7  Four 6  Five 0  Six 1  Seven 0  Eight+ 1 |
| **Household income (per month)** | **Region in the UK** | **Chronic illness** |
| ≤ £2,000 1  ≤ £3,000 3  ≤ £5,000 5  ≤ £6,000 3  ≤ £9000 1  ≤ £12,000 1  Student 1  Prefer not to say 2  Unsure 3 | South West 2  South East 1  East of England 1  London 7  East Midlands 1  Yorkshire & the Humber 2  North West 1  Wales 3  Scotland 3 | Yes 11  No 9  Prefer not to say 1 |
|  |  | **Caregiver** |
|  |  | Yes 4  No 15  Prefer not to say 1 |
|  |  | **Relationship status** |
|  |  | Single 8  In a relationship 2  Married/Civil Partner 11 |

**TABLE b.** Appointment details of participants.

| **Type** | **Setting** | **Reason** | **Duration** |
| --- | --- | --- | --- |
| In person 17  Video Call 3  Phone Call 1 | GP surgery 9  Mental Health Service 2  Hospital (inpatient) 2  Hospital (outpatient) 5  My own home 3 | Mental health 4  Physical health 7  *(Interviewees described physical health as a long-term or chronic condition during interview 7)* | 10-30 minutes 16  30 mins- 1 hour 2  1-2 hours 1  Unclear 2 |

**TABLE c.** Characteristics of healthcare professional rated by participants.

| **Known well to participant** | **Same age as participant** | **Same gender as participant** | **Same ethnicity as participant** | **Profession** |
| --- | --- | --- | --- | --- |
| Not at all well 7  Not well 5  Slightly well 2  Quite well 5  Very well 2 | Yes 2  No 19 | Yes 10  No 11 | Yes 9  No 12 | Doctor 11  Nurse 3  Midwife 2  Mental Health prof. 4  I do not know 1 |

**APPENDIX 5: Study 2 survey full demographics**

**TABLE d.** Demographics of participants.

| **Gender** | **Ethnicity** | **Age** |
| --- | --- | --- |
| Female 222  Male 85  Other 3  Prefer not to say 1 | White 294  Mixed 8  Asian or Asian British 6  Black or Black British 2  Other Ethnicity 1 | 18-24 29  25-34 63  35-44 36  45-54 36  55-64 59  65+ 88 |

**TABLE e.** Appointment details of participants.

| **Type** | **Setting** | **Reason** | **Length** |
| --- | --- | --- | --- |
| Face-to-face (PPE*) 129  Face-to-face (no PPE*) 11  Phone call 134  Video call 37 | Community venue 4  GP 66  Hospital inpatient 5  Hospital outpatient 58  Mental health service 24  Own home 125  Other 29 | Admin issue 2  Mental health 93  Physical health 140  Social problem 0  Other 76 | <10 mins 93  10-30 mins 136  30-60 mins 63  1-2 hours 16  2-5 hours 2  5+ hours 1 |

*PPE = Personal Protective Equipment

**TABLE f.** Characteristics of healthcare professional rated by participants.

| **Known well to participant** | **Same age as participant** | **Same gender as participant** | **Same ethnicity as participant** | **Profession** |
| --- | --- | --- | --- | --- |
| Not at all well 124  Not well 44  Quite well 59  Slightly well 56  Very well 28 | Yes 68  No 202 | Yes 173  No 131 | Yes 197  No 69 | Doctor 126  Health Care Assistant 2  Mental Health Nurse 53  Nurse 80  Nutritionist 4  Occupational Therapist 4  Paramedic 1  Physiotherapist 7  Administrator or  receptionist 4  Other 26 |

**APPENDIX 6: Item reduction additional analyses**

*Analyses of 14-item CHIA plus removal of item 14.*

**TABLE g.** Survey – Analyses of 14-item CHIA plus removal of item 14.

|  | **% NA’s** | **Inter-item correlations** | **IDI** | **Factor loading** | **Item fit (*p* value)** | **Local dependency** |
| --- | --- | --- | --- | --- | --- | --- |
| 1. The things that matter most to me were understood. | 3.54% | 0.76-0.87 | 5.17 | 0.92 | .000 |  |
| 2. I was taken seriously. | 0.96% | 0.77-0.88 | 6.70 | 0.93 | .044 | 0.225 (with Q4) |
| 3. The staff member showed me they had listened to me (e.g., by what they said or what they did). | 1.93% | 0.79-0.86 | 6.37 | 0.93 | .000 | 0.287 (with Q13) |
| 4. The staff member made time for me. | 0.64% | 0.73-0.86 | 4.89 | 0.90 | .000 |  |
| 6. I was given clear answers to my questions. | 6.75% | 0.78-0.86 | 4.87 | 0.91 | .000 |  |
| 7. The staff member explained things to me in a way that I understood. | 2.25% | 0.70-0.81 | 3.48 | 0.84 | .026 |  |
| 9. The staff member acted in my best interests. | 3.22% | 0.76-0.88 | 6.46 | 0.94 | .040 | 0.348 (with Q18) |
| 11. I was involved as far as possible in all decisions about my care. | 6.11% | 0.70-0.82 | 3.62 | 0.85 | .006 |  |
| 13. The staff member showed that they genuinely cared. | 0.64% | 0.76-0.89 | 6.37 | 0.94 | .000 | 0.208 (with Q2) |
| 15. The staff member showed that they could see things from my perspective. | 4.82% | 0.74-0.88 | 5.33 | 0.92 | .015 |  |
| 18. I trusted the staff member. | 0.64% | 0.76-0.84 | 5.31 | 0.90 | .035 |  |
| 19. The staff member treated me with respect. | 0.64% | 0.74-0.87 | 5.71 | 0.92 | .000 | 0.227 (with Q6) |
| 21. The staff member treated me with kindness. | 0.64% | 0.73-0.89 | 5.52 | 0.91 | .058 | 0.293 (with Q3) |

** Note: The item numbering from the original 21-item is retained in this table to make comparison with table 3 easier.*

***NA = not applicable, IDI = item discrimination index.*

**APPENDIX 7: Final 14-item CHIA**

**Compassionate Healthcare In Action (CHIA) Measure**

The aim of these questions is to try to find out what a recent experience of healthcare has been like for you, what was said and done, and how you experienced the care.

We are interested in your experiences of receiving care for physical and/or mental health needs. Please think about a recent encounter you had with a member of staff in a healthcare setting (e.g., at a hospital, your GP surgery, or at home).

Please read the statements below and rate how closely these statements fit with your recent experience of care from this staff member.

|  | Not at all | | | | Completely | | |
| --- | --- | --- | --- | --- | --- | --- | --- |
| 1. I was taken seriously. | 1 | 2 | 3 | 4 | 5 | 6 | 7 |
| 1. The staff member showed me they had listened to me (e.g., by what they said or what they did). | 1 | 2 | 3 | 4 | 5 | 6 | 7 |
| 1. The staff member showed that they could see things from my perspective. | 1 | 2 | 3 | 4 | 5 | 6 | 7 |
| 1. The things that matter most to me were understood. | 1 | 2 | 3 | 4 | 5 | 6 | 7 |
| 1. The staff member made time for me. | 1 | 2 | 3 | 4 | 5 | 6 | 7 |
| 1. The staff member treated me with respect. | 1 | 2 | 3 | 4 | 5 | 6 | 7 |
| 1. The staff member showed that they wanted to help me. | 1 | 2 | 3 | 4 | 5 | 6 | 7 |
| 1. The staff member showed that they genuinely cared. | 1 | 2 | 3 | 4 | 5 | 6 | 7 |
| 1. The staff member explained things to me in a way that I understood. | 1 | 2 | 3 | 4 | 5 | 6 | 7 |
| 1. I was given clear answers to my questions. | 1 | 2 | 3 | 4 | 5 | 6 | 7 |
| 1. I was involved as far as possible in all decisions about my care. | 1 | 2 | 3 | 4 | 5 | 6 | 7 |
| 1. The staff member acted in my best interests. | 1 | 2 | 3 | 4 | 5 | 6 | 7 |
| 1. I trusted the staff member. | 1 | 2 | 3 | 4 | 5 | 6 | 7 |
| 1. The staff member treated me with kindness. | 1 | 2 | 3 | 4 | 5 | 6 | 7 |

**Administration Instructions**

**When to use the CHIA**

The CHIA has been developed for use in physical and mental health consultation style and regular appointments lasting 8 minutes or longer. It has been developed specifically for use with adult populations (18+ years) when rating an appointment in which they are the primary patient/service user. This means it is not validated for use with children or for appointments where the adult is acting as a carer or parent and therefore are not the primary patient/service user.

This metric is only for use in consultation style appointments as routine checks (e.g. blood collection, flu vaccinations etc.) may not provide sufficient contact with professionals to be able to score the full range of questions in the metric. Similarly, appointments lasting less than 8 minutes will be unlikely provide sufficient content for the healthcare professional to be fairly scored.

**How to administer the CHIA**

This measure is intended to be a helpful adjunct to patient care. It should be administered in a situation where people do not feel pressured to respond in a certain way (e.g. people being able to take the measure away and respond or to be administered by a different member of staff to the person the interaction relates to). It could also be used as a basis for discussion with a member of staff. The intention is to be helpful for staff to improve their care, but working conditions also need to support compassionate care, and the system around the staff is important as well as individual staff behaviour. Therefore, it is suggested that scores from this metric are used in conjunction with a review of systemic practices that could support or be barriers to compassionate care.

**Scoring Instructions**

To calculate the total CHIA score, sum up each of the individual item scores. Higher scores indicate a greater perception of compassionate care received.

**APPENDIX 8: Study 3 survey full demographics**

**TABLE h.** Demographics of participants.

| **Sex** | **Ethnicity** | **Religion** |
| --- | --- | --- |
| Male 61  Female 231  Prefer not to say 4  Other 1  N/A 4 | Asian/Asian British 34  Black/African/Caribbean/Black British 18  White 225  Mixed/Multiple Ethnic Groups 12  Other Ethnic Group 9  N/A 3 | No religion 154  Christian 115  Buddhist 3  Hindu 8  Jewish 1  Muslim 5  Sikh 3  Any other religion 8  N/A 4 |
| **Gender different from sex assigned at birth** |  |  |
| Yes 16  No 282  N/A 3 |  |  |
| **Age** | **Sexual orientation** | **Current employment** |
| 18-24 111  25-34 47  35-44 30  45-54 50  55-64 37  65+ 23  N/A 3 | Straight or heterosexual 243  Gay or Lesbian 8  Bisexual 30  Pansexual 5  Asexual 8  Other sexual orientation 4  N/A 3 | Employee 143  Self-employed 31  Retired 24  Studying 72  Long-term sick or disabled 15  Any other paid work 2  None of the above 12  N/A 2 |
| **Highest level of academic attainment** | **Disability** | **Region in the UK** |
| No qualifications 5  GCSEs or equiv. 19  AS, A Level or equiv. 38  NVQ or equiv. 7  Degree or equiv. 127  Postgraduate degree 106  N/A 3 | No disability 218  Learning disability 12  Mobility impairment 14  Hearing loss 9  Visual impairment 9  *Neurodiversity:*  ADHD 14  Autism 40  Dyslexia 3  Tourettes Syndrome 1  Suspected/undiagnosed 5  Other neurodivergence 2  Unclear 13 | South West 101  South East 42  East of England 20  London 46  West Midlands 10  East Midlands 9  Yorkshire & the Humber 7  North West 10  North East 5  Northern Ireland 3  Wales 10  Scotland 7  N/A 31 |
| **Household income (per month)** | **Relationship status** | **Chronic illness** |
| < £2,000 104  < £5,000 34  < £8,000 16  > £8,000 20  Student 13  Unsure/  Prefer not to say 14 | Single 110  In a relationship 66  Married/Civil Partner 111  Divorced/Person whose Civil Partnership is dissolved 5  Widowed/surviving Civil Partner 6 | Yes 62  No 225  Prefer not to say 8 |
|  |  | **Caregiver** |
|  |  | Yes 18  No 271  Prefer not to say 4 |
| **Household size** | **Contact with loved ones** | **Frequency of Monthly Social Activities** |
| One 41  Two 87  Three 60  Four 61  Five 35  Six 6  Seven 3  Eight+ 3  N/A 5 | Never 7  Rarely (1-3x per month) 37  Regularly (1-2x per week) 87  Often (3-6x per week) 60  Everyday 94  N/A 16 | Once a week 114  Twice a week 70  Three times a week 41  Four times a week 24  Five times a week 17  Six times a week 0  Seven times a week 11  More than 7 times 2  Unclear 8  N/A 14 |

**TABLE i.** Appointment details of participants.

| **Type** | **Setting** | **Reason** | **Duration** |
| --- | --- | --- | --- |
| In person 239  Video Call 14  Phone Call 48 | GP surgery 152  Mental Health Service 18  Hospital (inpatient) 9  Hospital (outpatient) 49  Community venue 12  My own home 30  Other 31 | Mental health 56  Physical health 199  Social problem 1  Administrative issue 5  Other 40 | <10 minutes 40  10-30 minutes 202  30 mins- 1 hour 40  1-2 hours 5  2-5 hours 3  5 hours plus 3  N/A 8 |

*PPE = Personal Protective Equipment

**TABLE j.** Characteristics of healthcare professional rated by participants.

| **Known well to participant** | **Same age as participant** | **Same gender as participant** | | **Same ethnicity as participant** |
| --- | --- | --- | --- | --- |
| Not at all well 147  Not well 56  Slightly well 44  Quite well 38  Very well 16 | Yes 65  No 236 | Yes 193  No 108 | | Yes 170  No 131 |
| **Profession** | | | | |
| Doctor 155  Nurse 61  Midwife 3  Mental Health Professional 28  Occupational Therapist 7 | Physiotherapist 12  Nutritionist 0  Healthcare Assistant 1  Pharmacist 13  Paramedic 1 | | Receptionist/administrator 5  Cleaner 1  Porter 0  I do not know 1  Other 13 | |

**APPENDIX 9: Ceiling effects of the other compassionate care measures in study 3**

| **Compassionate Care Measure** | **Ceiling Effects (%)** |
| --- | --- |
| CHIA | 29 - 54 |
| SCCS | 13 - 26 |
| FIT | 17 - 28 |
| CARE | 19 - 28 |
| SCQ | 21 - 33 |
